# An exploratory study of communication training for Chinese medicine practitioners in Hong Kong to integrate patients’ conventional medical history

**DOI:** 10.1186/s12906-022-03811-x

**Published:** 2023-01-12

**Authors:** Jack Pun, Brandon Kong

**Affiliations:** Department of English, City University of Hong Kong, 83, Tat Chee Avenue, Kowloon Tong, Hong Kong SAR, China

**Keywords:** Traditional Chinese medicine, Communication training, Integrated medicine

## Abstract

**Background:**

Despite Traditional Chinese medicine’s (TCM) historical roles in Chinese society, few research has been investigated the nature of TCM practitioner–patient interactions. Improved communication skills among TCM practitioners will result higher-quality interactions and better clinical outcomes.

**Methods:**

To investigate the changes in TCM practitioners’ communication practices after communications training focused on promoting their awareness of integrating a patient’s medical history from conventional medicine in TCM treatment, Eight registered Cantonese-speaking TCM practitioners in Hong Kong were randomly recruited from local clinics and randomised into control (*n* = 12) and experimental groups (*n* = 12), with a total of 24 consultations. The experimental group was given training focused on patient-centred communication, with an internationally recognised and communication framework validated in global consultation settings (i.e. the Calgary-Cambridge Guide) on how to take a patient’s medical history from conventional medicine and communicate diagnosis and treatment plans. Consultations before and after training were audio-recorded and rated. The efficacy of the training was evaluated by comparing the two groups before training (pre-test), immediately after training (post-test) and after a 3-month delay (delayed post-test). Using validated scales, the primary outcomes were measured for the practitioners’ clinical communication skills and the quality of interactions.

**Results:**

The communication training significantly improved the TCM providers’ patient-centred communication and communication proficiency. The results indicate that the team developed an effective communication model for integrating TCM and conventional medicine in Hong Kong. The framework helps trained TCM practitioners to integrate their patients’ conventional medical history when delivering patient care. The findings shed light on how interpersonal relationships between TCM practitioners and patients can be constructed after communication training to better care for patients’ psychological concerns in addition to their physical needs.

**Conclusion:**

Trained TCM practitioners can provide an integrated model that takes patients’ conventional medical history into account when delivering a holistic patient-centred care. The findings can enhance our understanding of better ways to train the future TCM practitioners and to develop a continuing professional training for the current TCM practitioners to expand our understanding of TCM communication in acute clinical contexts and, thus offer a firm evidence-based foundation upon which to develop communication strategies that improve their clinical cpractices.

## Background

Traditional Chinese medicine (TCM) is in relatively high demand in Hong Kong [1–6]. Individuals’ attitudes towards TCM are a significant factor in their choice to use TCM [7]. The reported effectiveness of TCM treatments for COVID-19 implies a generally favourable attitude toward TCM among Chinese communities [8–10]. This may also explain the overall recognition of TCM and suggests that it may increase further in popularity in the coming years [11, 12]. TCM is holistic in nature, as all organs and systems are taken into consideration for an individual’s healing and recovery [13]. Furthermore, the holistic recovery of a patient involves such elements as individual experiences, daily activities, surrounding environments and the services and facilities they interact with on their recovery journey [6]. The holistic perspective may also contribute to the understanding of how TCM treatments of a patient’s physical health can help with mental illnesses such as depression [14, 15], as there is an active awareness of the complementary and integrative natures of physical and mental health [16]. This practice and philosophy of TCM can be argued as a contrastive approach to the conventional biomedical view, within which there is a stronger tendency to focus on the aetiological and diagnostic natures of patients’ illnesses, which are built upon empirical and systematic evidence from the scientific literature [6, 17]. A more extreme view that has been postulated is that ‘Chinese medical theory bears no resemblance to biomedicine’ ([18], p.101). Despite the effectiveness of TCM treatments, conventional medicine has undoubted value, as it is the dominant medical practice in many parts of the world. The perceived importance of conventional medicine even in China can be found in the debate over whether TCM or conventional medical treatments are more effective in cases of cancer^6^. The data collected from conventional medicine have also proven extremely useful in drawing out the causes along with the effects and symptoms of illnesses [6, 17].

In considering a patient’s health and recovery from illness, the medical staff, procedures and services, along with the ingredients and substances used for treatment, are important but not the only factors. For instance, part of the reason that people in mainland China and Hong Kong seek TCM treatments might be that traditional medicine can be strongly associative with one’s own culture and identity [17, 18]. Furthermore, the language and discourses used to construct and maintain relationships in practitioner–patient interactions are crucial to patients’ overall consultation experience and recovery [3–6, 19–25]. For example, researchers observed that the relatively balanced conversational turn-taking between TCM practitioners and their respective patients reflects the holistic care of a patient’s physical and psychological health. Distinctions between TCM and conventional medicine have been observed within practitioner–patient communication and the analyses of their ongoing interactions.

In research on the TCM context, the communication between TCM practitioners and their patients has been shown to be generally favourable. For example, Wang argued that TCM practitioners can provide more interpersonal support than their conventional biomedical counterparts because of superior communication skills [26]. A further benefit of TCM is that patients often consult the same practitioner, which promotes continuity of care and practitioner–patient relationship-building and is not as common in conventional medicine consultations [17]. The improved relationship-building and support imply a better understanding of patients’ conditions among TCM practitioners, which could lead to better decisions and more effective treatments [4–6]. However, one of the major findings of Chung and colleagues was that although patients perceived TCM practitioners as better listeners than their conventional medicine counterparts, they could also appear less respectful to the patient – the probable causes of which are the consultation setting and insufficient formal communication training [27]. It would therefore not be a fair assessment to simply state that the communicative practices in conventional medical environments are not as effective as those in TCM consultations.

Many studies have stressed the importance of analysing the discourses and communicative praxis in various clinical and medical settings [28–31]. Studies of effective healthcare communication in these settings have examined improving the outcomes of nurse–patient interactions [32–36], understanding potential communication problems in genetic and medical consultations [37], increasing awareness of patients’ needs and supporting them in their interactions and involvement [21], and how trust is promoted and maintained among healthcare workers [38]. Therefore, notwithstanding the ostensible contrasts between TCM and conventional medicine, we believe in the integration of the knowledge, philosophies, and practices of conventional medicine and TCM to effectively treat patients. This is not a novel proposition, as researchers have reported on observations of patients consulting both TCM and biomedical treatment providers [2, 39, 40]. The application of both TCM and conventional medicine has produced positive clinical outcomes [8]: for example, Liu et al. reported that TCM had helped patients undergoing radiotherapy and chemotherapy [41].

Despite these studies showing the clinical effectiveness and benefits of integrating TCM and conventional medicine, there is a paucity of research into the communicative and discursive elements involved, especially in communities where TCM is popular like Hong Kong [42]. Accordingly, this article evaluates the outcomes for TCM practitioners who have undergone an intervention programme based on the integration of conventional medicine, TCM and healthcare communication training. We maintain that such an intervention can improve TCM practitioners’ communication skills, relationship-building with patients and treatment decision-making. Generally, a TCM medical history which integrates a patient’s conventional care is defined as an inquiry into a patient’s past medical history from their conventional treatments, past surgical history, family backgrounds, allergies, and medications that the patient is taking from their conventional medicine. To be more precise, this study advocates taking conventional medical treatments into consideration when discussing treatment plans and diagnoses.

There is a growing research evidence that TCM practitioners provides more interpersonal support to patients than conventional medicine because TCM practitioners perhaps acquire better communication skills and communicate better with patients [26, 43]. In Chung et al.’ s study, patients report that they prefer TCM when comparing with Western Medicine (WM) due to the fact that they feel TCM practitioners have better listening skills [27, 44]. This is recognised by study like Jagosh et al*.* who report that by being instrumental to building a sustainable doctor-patient partnership that can enhance the patient’s healing process [45]. The findings of Chung et al.’s study are important to inform our understanding about the exploration of what is feasible in term of doctor-patient communications in a cross-cultural, and cross-context (ie. TCM vs WM), particularly for TCM communication [44]. The Chung study was the first to research clinical communication and patients’ perceptions in both TCM and conventional medical practices, observing that patients were more satisfied with the listening skills of TCM practitioners than conventional medical doctors. According to the authors, patients received conventional medicine who tended to be dissatisfied with the quality of their doctor-patient communications [26]. The importance of good doctor-patient communication, however, has been widely acknowledged given that it can affect medical outcomes like patient compliance to treatment, satisfaction, ‘symptom resolution’, complaints and the control of pain [46–50].

To the best of our knowledge, studies comparing patients’ perceptions of TCM and conventional medicine in China and the U.S. suggested that TCM practitioners had better communication skills than their WM counterparts [26, 27, 44, 46–50]. The results suggest that the long-term relationships between TCM practitioners and their patients have a positive effect on doctor-patient communications [45]. Effective doctor-patient communication refers to medical interaction in which information is exchanged and a positive interpersonal relationship between doctor and patient is concurrently built. The relationship is one in which the patient is involved in his or her own care in collaboration with the doctor [51].

By reviewing the literature on healthcare in Hong Kong, analysing the Hospital Authority risk analysis reports and recent media coverage on hospital crises, in addition to undertaking a pilot project in a major public hospital for the Hospital Authority, we believe that effective health communication is critically important to Hong Kong and that far-reaching research should be undertaken to produce a well-informed communication framework for education and professional development, especially for TCM practitioners. In most English-speaking countries or contexts where English is being used in clinical setting, established communication protocol such as the Calgary-Cambridge Guides has been adopted for teaching clinicians about ways to communicate with patients [52]. However, in our understanding of the existing literatures, there is no similar models currently presented for non-English-speaking culture and very limited work has been explored the effective way of implementing a translated Western models of communication framework to enhance TCM practitioners for their clinical communication skills. The ample number of studies have reported on the differences in clinical communication between English and non-English speaking contexts [53, 54], thus suggesting that the inappropriateness of ‘borrowing and adopting’ the desired Western model in Chinese contexts.

To address this gap, a few scholars have modified certain communication frameworks like the SEGUE (set the stage, elicit information, give information, understand the patient’s perspective and end the encounter) framework [55]. Deriving from Western ideologies, the SEGUE framework aims to improve doctors’ communication skills and highlight the cultural differences between Chinese contexts and Western contexts [56]. However, the framework does not effectively adapt to particular Chinese cultural factors like collectivism. Moreover, it fails to offer any communication patterns of how medical students with limited clinical experience respond to patients emotional needs stemming from their cultural backgrounds.

Communication training sessions are often limited in Chinese contexts as medical schools, clinics and hospitals rarely address the importance of sociocultural factors in medical communication. As the result, clinicians are merely trained to communicate direct medical information instead of addressing patients’ emotional needs [57]. There are no standardised communication protocols that integrate local culture into the training in understanding and interpreting medical encounters. Therefore, doctor-patient relationships are highly at risk. In order to fill such a research gap, the study investigated the changes in TCM practitioners’ communication practices after adopting the Calgary-Cambridge Guide on how to take a patient’s medical history from conventional medicine and communicate diagnosis and treatment plans.

The hypothesis is that TCM practitioners who discuss their conventional medical history with patients will perform better in terms of their clinical communication skills and quality of interactions, resulting in better patient outcomes such as increased levels of patient satisfaction and treatment adherence. In relation to this hypothesis, the study analyses effective communicative practices between TCM practitioners and their patients to determine ways to integrate their conventional medical history into diagnosis and treatment. The analysis of these conversations supports the development of an integrative model for aiding TCM practitioners to make treatment decisions for their patients who also receive conventional medical care.

The analysis of the data collected from TCM practitioners who have undergone communication training addresses the following research questions. (1) What knowledge is gained, changed, or lost in the flow of information during TCM medical consultations when patients provide medical information from their conventional medicine doctors? (2) How do TCM practitioners prompt patients to articulate relevant information when accounting for the patients’ perspectives on their conventional medical encounters? (3) What strategies do TCM practitioners use during consultation to understand the patients’ perspectives on their conventional-based medical experiences. The details of the intervention and data collection are elaborated in the next section.

## Methods

As this study is based upon both the application and evaluation of communication training in promoting TCM practitioners to integrate patients’ conventional medical history in their TCM care, the methods and the data collection process comprised multiple stages: (1) development and validation of the communication training; (2) intervention; and (3) video-recorded ethnographic observation of TCM practitioner–patient interactions.

In the development and validation stage, three TCM practitioners from the Chinese University of Hong Kong (CUHK) and Hong Kong Baptist University (HKBU) formed an expert focus group to discuss and validate the analytical framework and adapt them to the Hong Kong context for content validity. Any discrepancies in the evaluative comments of the first and second experts were resolved through negotiation with the third expert. The analytical framework for analysing the communication between TCM practitioners and patients were all checked for content validity using input from the three health experts, and the subsequent calculations were based on the content validity index.

After the validation, the authors recruited participants for the intervention. The participants were TCM practitioners officially registered by the Chinese Medicine Council of Hong Kong (https://www.cmchk.org.hk/cmp/eng/#main_rdoctor_choice.htm) and their patients. The other inclusion criteria were that (1) the practitioners and patients were Chinese adults and able to communicate in Cantonese and (2) the patient had at least one chronic disease and had received conventional medical treatment in either private and public hospitals or clinics within the 12 months prior to their TCM visit. Patients were excluded if their TCM visit was not related to the condition that prompted their previous use of conventional medicine. Based on these criteria, eight practitioners and eight patients were qualified and willing to participate in the intervention.

The practitioners and their patients were randomly allocated with a 1:1 ratio to either the experimental group (i.e. the group that would receive the communication training) or the control group. After the allocation, the clinician participants were asked to complete a background survey on their background, experience, and patients’ demographics. For the experimental group, the practitioners and patients underwent a 3-h communication intervention based on the Calgary-Cambridge Guide for teaching clinical communication [52]. To fit the intervention to the Hong Kong context, modifications were made based on a pilot study that explored Chinese patients’ communication needs in TCM consultations^3^ and previous projects in accident and emergency services, clinical handover at nursing and an intensive care unit [58–61]. Each participant in the training was also provided with a theory and practice manual and conducted a role-play exercise with simulated patients in a tutorial setting with individual feedback. The TCM practitioners in the experimental group perform normal TCM care to patients but they asked patient’s medical history from their conventional care. A history taking stage in TCM will include an inquiry into a patient’s past medical history from their conventional treatments, past surgical history, family backgrounds, allergies, and medications that the patient is taking from their conventional medicine. To be more precise, this study advocates taking conventional medical treatments into consideration when discussing treatment plans and diagnoses. The participants allocated to the control group did not undergo the training, and the patients therefore received the default standard of care provided by their respective practitioners.

To address the research questions, these activities were followed by ethnographic observations of TCM practitioner–patient consultations. After obtaining clear written consent from the practitioners and patients, research assistants audio-recorded all the medical consultations through to the discharge process and final practitioner–patient consultations. Verbal practitioner–patient interactions were collected and catalogued by the researchers. Given that there were eight practitioners (four in the control group and four in the experimental group) involved in the study, the ethnographic recordings of pre-test (before training), post-test (after training), and delayed post-test (three months after training) consultations made for a total of 24 consultations in the dataset. To assess the outcomes of the experimental and control groups, our analysis of the consultations proceeded in three main steps: macro-analysis, micro-analysis and scoring according to the Calgary-Cambridge Guide.

### Macro-analysis

For the macro-analysis, we adapted the methodology proposed by Tsai [62], with the utterances initiated by the practitioners assigned into three main categories and six commonly used formats (see Tables 1 and 2; the content of all of the utterance formats can include the three categories of general, biomedical and psychosocial questions). The categories can be seen as the content of the utterance, whereas the format denotes the discursively salient markers and the manner in which they are articulated. This method can turn the practitioners’ utterances into a useful framework for analysing the interaction between patient and doctor [4].

**Table 1:** Categories and semantic functions of questions used in doctor–patient interactions (adapted from Tsai [62])

Categories	Semantic functions	Example
1. General questions	Cover all patient’s problems	What problem brings you here today?
2. Biomedical questions	Cover patient’s biological or physical states	What is your discomfort?
3. Psychosocial questions	Cover patient’s psychological or social problems such as lifestyle, social events, concerns, expectation, desire	What changes have there been in your life recently?

**Table 2:** Format of questions used in doctor–patient interactions (adapted from Tsai [62])

Format	Example
1. Wh-Q	What pains are you suffering from?
2. Y/N Q	Is there anything I can do for you today?
3. Indefinite enumeration	Do you have anything like hypertension, diabetes?
4. Sentential fragment	Any other problems?
5. Statement	Tell me all the problems you have
6. Continuer marker	And?

### Micro-analysis

**Fig. 1:**
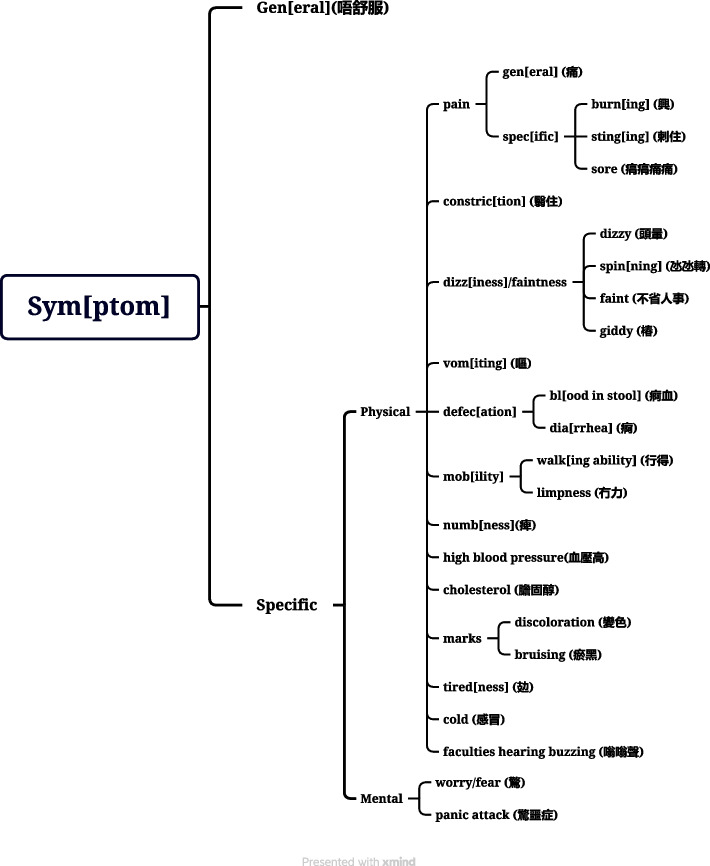
Experiential dimension: Lexical network (adapted from Slade et al. [63])

The micro-analysis was based on the methodology proposed by Slade et al., who stated that communication has both interpersonal and experiential dimensions [63]. The interpersonal dimension of the TCM consultations is analysed in terms of moves and speech functions (see Tables 3 and 4), and the experiential dimension is analysed in terms of lexis and the lexical network (see Fig. 1). In their words ([63], p.240),

the move and speech function analysis provide a perspective on interpersonal patterns in the discourse, showing how the consultation develops in terms of the speech roles taken up by doctor and patient. The lexical analysis provides an experiential perspective on the discourse, showing how medically related information unfolds during the course of the consultation.

**Table 3:**
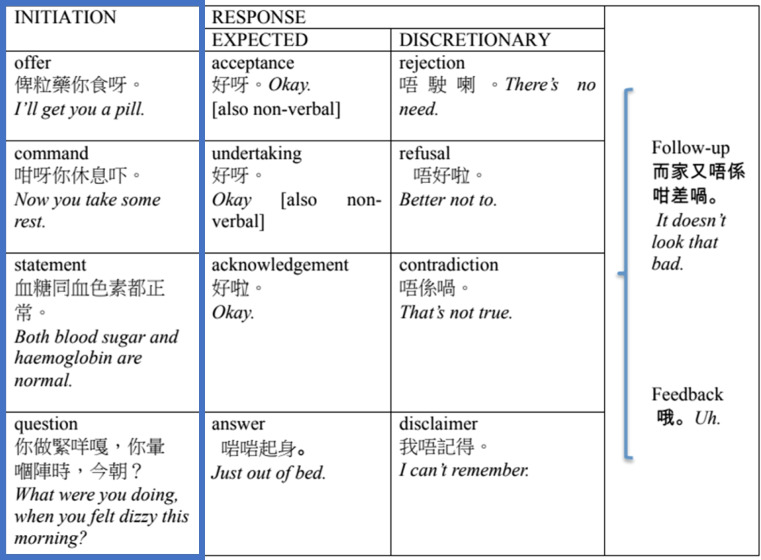
Interpersonal dimension: Moves in an exchange (adapted from Slade et al. [63])

**Table 4:**
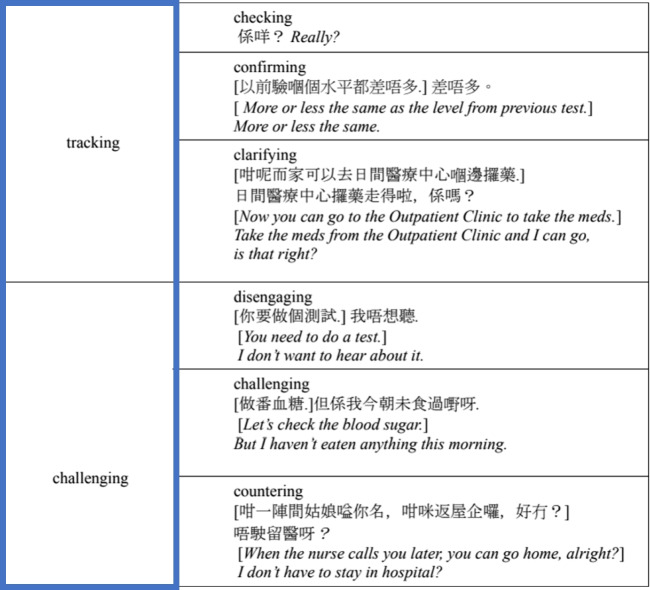
Interpersonal dimension: Tracking and challenging moves in an exchange (adapted from Slade et al. [63])

This two-part micro analytical framework was adopted for this study to thoroughly assess the communication of the TCM practitioners.

In analysing the moves and speech functions, the emphasis was placed on the initiation of the TCM practitioners rather than the responses of the patients, as we wanted to explore in detail the communication skills demonstrated by the practitioners. To clarify the terminology, Slade et al. [63] explained that

An exchange consists of one or more moves, prototypically an initiating move and a responding one. A move is a unit of interaction where the speaker moves the dialogue forward by choosing a speech function such as a question or a statement, taking on a speech role and assigning a complementary role to the addressee, e.g. questioner — expected answerer. Two or more moves may function together in the development of the exchange that they are part of, forming a move complex; for example, a question may be preceded by a statement giving some background to the question.

In terms of lexis, Slade et al. identified lexical items related to three major aspects of the field of discourse within the HEPC (History and Exploration of Presenting Condition) stage. These aspects are incident, history and symptoms: respectively, events related to the incident leading to the patient’s attendance at the consultation, medical conditions and medication relevant to the patient’s medical history, and descriptions of the patient’s medical symptoms. This study followed the coding method of Slade et al., who used the lexical network displayed in Fig. 1.

Still in relation to lexis, two kinds of shifts were introduced in the work of Slade et al. [63]: the minor shift and the major shift. A minor shift is a shift within a major aspect – for example, from a move ‘symptom: specific: headache’ to a move ‘symptom: specific: dizzy’. A major shift is a shift between two major aspects – for example, from ‘incident: car crash’ to ‘history: medical treatment’.

For the qualitative analysis, the Calgary-Cambridge Guide – ‘a well-known approach to teaching and training clinical communication skills’ ([64], p.1) – was used to assess the communication skills of the practitioners. The applicability and widespread use of this guide in the health, clinical and medical fields make it a useful framework for the analysis in this study [65–67]. The Calgary-Cambridge Guide contains seven sessions and 73 statements, of which 67 were selected for scoring in the present study because the remaining six were related to non-verbal skills and preparatory work and thus could not be verified from the audio recordings. It is common for some of the communication scoring criteria to be absent or not able to be evaluated in consultation data [65].

## Results

### Results for all practitioners

**Table 5:** Percentages of questions asked by and conversational turns of practitioners^a^

	Pre-test	Post-test	Delayed post-test
**Control group (pseudo name)**			
TCM Practitioner 1	56	38	42
TCM Practitioner 2	91	83	63
TCM Practitioner 3	76	71	51
TCM Practitioner 4	60	61	60
**Experimental group (pseudo name)**			
TCM Practitioner 5	65	76	81
TCM Practitioner 6	31	60	41
TCM Practitioner 7	43	71	51
TCM Practitioner 8	38	71	52

^a^Percentages were preferred to absolute numbers in reporting the results because the latter approach would have created difficulties in comparing and contrasting consultations. As the length and number of turns of each consultation differed, the ratio of questions is more revealing than the absolute number of questions

Table 5 shows the numbers of questions asked by the practitioners in the control and experimental groups as percentages of the numbers of conversational turns they uttered. In the control group, practitioners Lam, Poon and Luo asked fewer questions in their respective post-test and delayed post-test consultations than in their pre-test consultations. The number of questions asked by practitioner Wang was virtually constant (post-test consultation questions increased by 1%). In the experimental group, all of the practitioners asked many more questions during their post-test consultations than during their pre-test consultations. Furthermore, the intervention appears to have encouraged the practitioners to explore patients’ information in more detail, given that all of the practitioners were still asking more questions in their delayed post-test consultations than in their pre-test consultations. However, with the exception of practitioner Kwan, there was a noticeable decrease in the number of questions asked from the post-test to the delayed post-test consultations, suggesting that the optimal effectiveness of the training was limited to a short period. Furthermore, the practitioners in the experimental group asked a much lower percentage of questions in their pre-test consultations than their control group counterparts, which may have skewed the results to some extent.

**Fig. 2:**
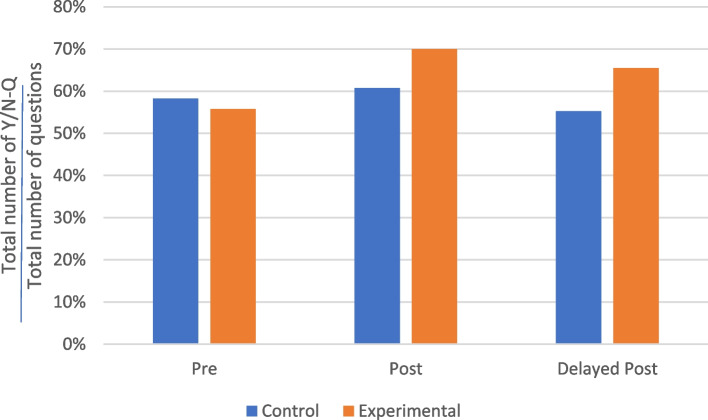
Proportions of Y/N-Q questions asked by the TCM practitioners before and after training

The most frequent question format used by the practitioners in both the control and experimental groups was Y/N-Q, which made up over half of all questions at all three stages (see Fig. 2). At the pre-test consultations, the practitioners in the control group asked slightly more Y/N-Qs than those in the experimental group. However, after the training, the practitioners in the experimental group asked more Y/N-Qs than those in the control group in both the post-test and delayed post-test consultations, with margin of 9% and 11%, respectively (70% vs 61% in post-test consultations; 66% vs 55% in delayed post-test consultations).

**Fig. 3:**
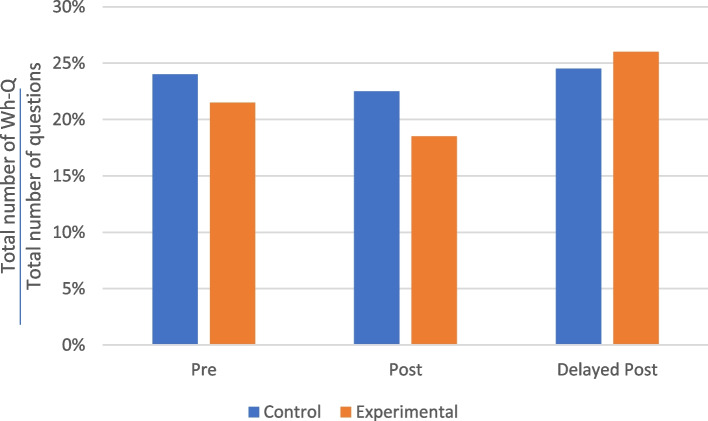
Proportions of Wh-Q questions asked by the TCM practitioners before and after training

The second most frequent question format used by the practitioners was Wh-Q (see Fig. 3), with this format making up an average of approximately 22% of all questions. Read alongside the frequency of Y/N-Qs shown in Fig. 2, this aligns with the findings of Slade et al. that the ratio of Wh-Qs to Y/N-Qs was higher among doctors from more conventional backgrounds [63]. On average, the practitioners in both the control and experimental groups asked fewer Wh-Qs in their post-test consultations than in their pre-test consultations but more Wh-Qs in their delayed post-test consultations than in their pre-test consultations.

**Fig. 4:**
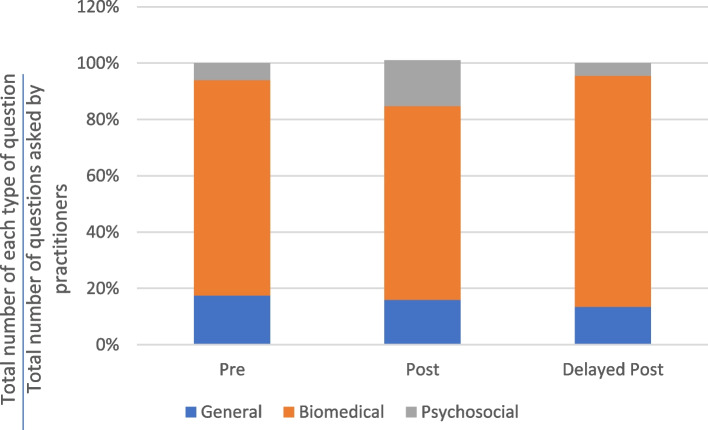
Distribution of major types of questions asked by TCM practitioners in the control group

**Fig. 5:**
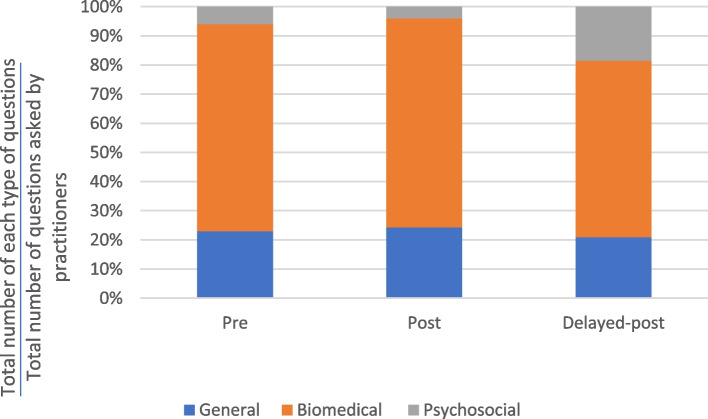
Distribution of major types of questions asked by TCM practitioners in the experimental group

Figures 4 and 5 show that most of the questions asked by the practitioners were biomedical questions related to the physical state of the patients. Across the consultation stages, the proportion of questions that were biomedical ranged from 62 to 82%. The varying distribution of question types across the different consultation stages suggests that the intervention had minimal impact on the types of questions asked by the practitioners.

The Calgary-Cambridge Guide scores are tallied and compared between the control and experimental groups in Table 6. The practitioners in the experimental group scored higher on average in both the post-test and delayed post-test consultations than those in the control group. However, although only one practitioner in the control group scored lower in the guide assessment in the delayed post-test consultation than in the pre-test consultation, this pattern was observed in two of the practitioners in the experimental group. Furthermore, the higher scores of three of the practitioners in the control group at the delayed post-test consultation than at the pre-test consultation suggests that the nature of the consultations was affected by other variables in addition to the intervention.

**Table 6:** Calgary-Cambridge Guide scores for practitioners during their consultations

	Pre-test	Post-test	Delayed post-test
**Control group**			
TCM Practitioner 1 (Lam)	32	35	29
TCM Practitioner 2 (Luo)	23	22	28
TCM Practitioner 3 (Poon)	18	44	37
TCM Practitioner 4 (Wong)	27	29	30
**Experimental group**			
TCM Practitioner 5 (Kwan)	24	48	34
TCM Practitioner 6 (Chung)	34	36	30
TCM Practitioner 7 (Man)	38	37	35
TCM Practitioner 8 (Wang)	22	31	39

**Table 7:** Number of practitioners who addressed patients’ conventional medical histories during consultations

	Pre-test	Post-test	Delayed post-test
Control group	3	1	2
Experimental group	3	3	2

Although the number of practitioners addressing patients’ conventional medical histories during consultations was the same in the pre-test and delayed post-test consultations, there was a relative consistency among the practitioners in the experimental group in incorporating this subject matter in the post-test consultation (See Table 7). Although these results need to be replicated to confirm their veracity, the intervention may have served as a reminder to the practitioners of the importance and value of exploring patients’ conventional medical histories.

The results presented in the tables and graphs above provide a broad overview of the interactions that the TCM practitioners had with their patients. For a better understanding of the interactions that took place during the consultations at a micro level, extracts from the consultations held by Kwan, one of the participants in the experimental group, are shown and analysed in the next section. Of the eight practitioners in this study, Kwan’s consultations revealed the most salient findings and themes.

### Case study of one practitioner (Kwan)

**Fig. 6:**
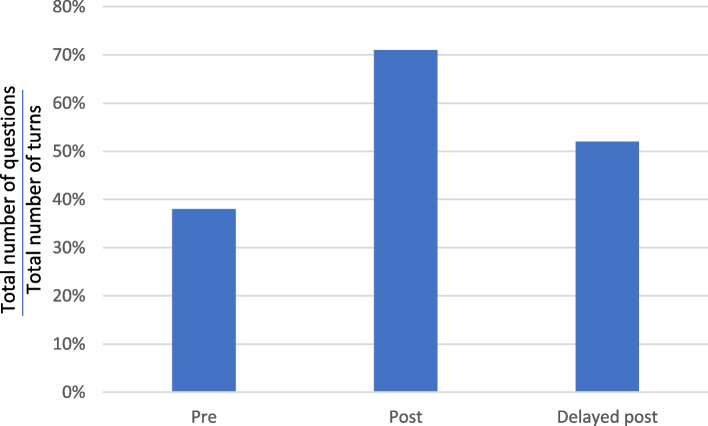
Questions as a percentage of turns – Kwan

Kwan was one of the four TCM practitioners in the experimental group. Kwan uttered 13, 42 and 23 turns^1^ in his pre-test, post-test and delayed post-test consultations, respectively. Figure 6 shows that the total percentage (number) of turns that involved asking questions increased significantly from 38% (5) in the pre-test to 71% (30) and 57% (13) in the post-test and delayed post-test consultations, respectively (See Fig. 6).

**Fig. 7:**
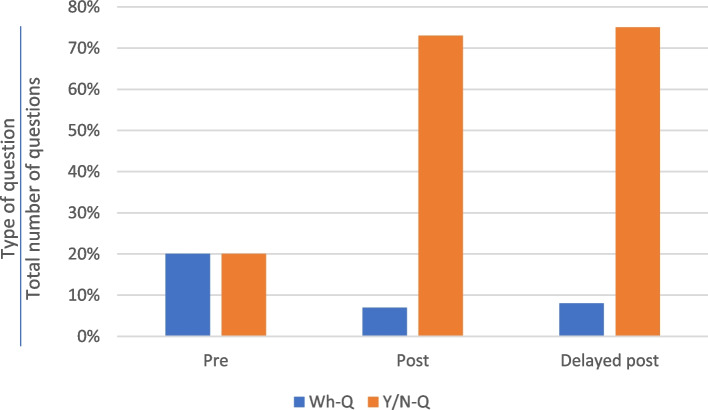
Distribution of Y/N-Q questions and Wh-Q questions – Kwan

As shown in Fig. 7, there were significant differences between Kwan’s three consultations in his use of Wh-Qs and Y/N-Qs. In the post-test and delayed post-test consultations, although Kwan asked fewer questions overall, he emphasised Y/N-Qs at the expense of Wh-Qs. Y/N-Qs constituted 73% and 75% of questions in the post-test and delayed post-test consultations, respectively, but only 20% in the pre-test consultation. Although it has been proposed that Wh-Qs can elicit more information than Y/N-Qs (Slade et al. [63]), the macro-analysis above indicates that the TCM practitioners in our study favoured Y/N-Qs over Wh-Qs.

**Fig. 8:**
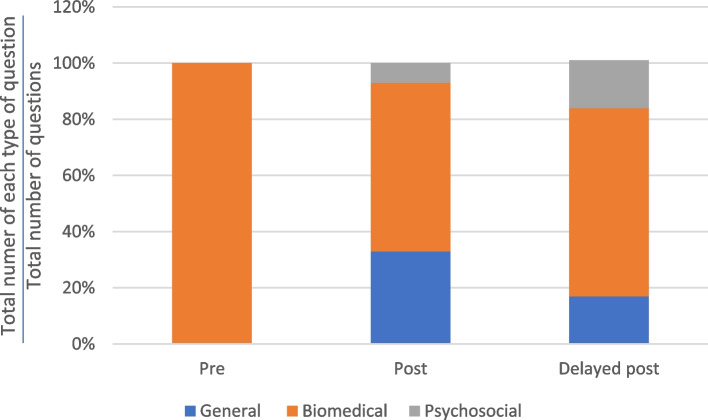
Distribution of the major types of questions – Kwan

As shown in Fig. 8, Kwan only asked biomedical questions in the pre-test consultation. After the intervention, Kwan used general and psychosocial question types. Kwan’s increased use of psychosocial questions from the post-test to the delayed post-test consultations suggests an increased and sustained awareness of the importance and value of this question type. In Kwan’s delayed post-test consultation, the proportion of general and psychosocial questions was 17% higher and that of biomedical questions was 33% lower than in the pre-test consultation.

The purpose of asking questions in a consultation is usually to acquire new information or clarify previously acquired information. According to the data on Kwan’s consultations presented above, we can predict that he achieved these goals in the post-test and delayed post-test consultations. Furthermore, as general and psychosocial questions are closely related to patients’ daily life information or medical history rather than their present physical state, we can surmise that Kwan was more active in collecting the patient’s daily life and medical information in the post-test and delayed post-test consultation than in the pre-test consultation. Integrating the three main types of question might also help practitioners to build a better rapport with their patients. Based on the distribution of question types in Kwan’s three consultations, he requested more kinds of information in the post-test and delayed post-test consultations, which implies a greater degree of holistic care through a more explicit concern with the psychosocial, lifestyle and experiential aspects of the patient.

**Fig. 9:**
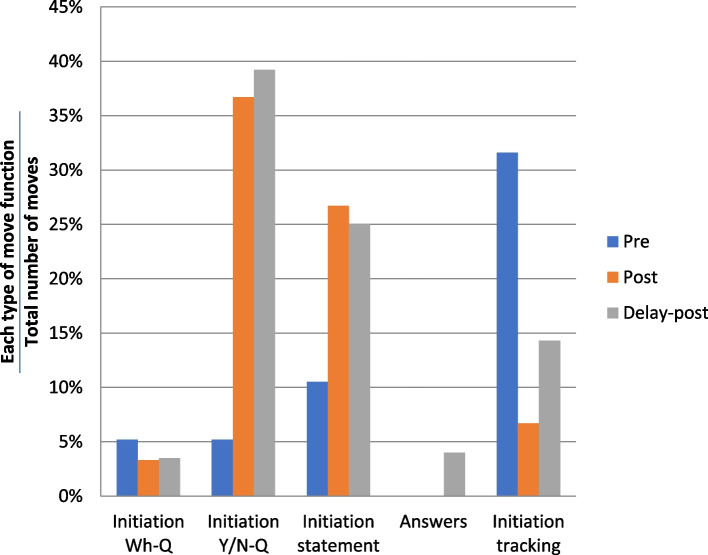
Types of move functions – Kwan

As shown in Fig. 9, Kwan significantly increased his use of Y/N-Qs and statements from the pre-test to the post-test and delayed post-test consultations. Statements aim to provide the patient with detailed information or explanations [62]. Kwan’s increased use of statements therefore strongly suggests that he provided more detailed information to the patient in the post-test and delayed post-test consultations. The much more frequent use of tracking moves in the pre-test consultation than in the two later consultations shows that Kwan mostly repeated and clarified what the patient said rather than initiating questions in the pre-test consultation.

## Discussion

This study examines the effect of including patients’ conventional medical history and treatment in the treatment plan and diagnosis on TCM practitioners’ clinical communicative skills. In three steps – a macro-analysis, micro-analysis and scoring on the Calgary-Cambridge Guide – the study analysed the effective communicative practices of TCM practitioners with their patients regarding the integration of their conventional medicine medical history at the stages of diagnosis and treatment planning. The study demonstrated that the incorporation of patients’ conventional medical history can lead to practitioners having a more holistic understanding of their patients’ conditions. Despite the intervention making no significant difference to the question types adopted by the practitioners during the consultations, the ratio of total number of questions asked and conversational turns of practitioners in the experimental group increased markedly from the pre-test consultations to the post-test consultations. However, the effect of the intervention declined over time. Furthermore, the practitioners in the control group mostly scored higher on the Calgary-Cambridge Guide in the post-test consultation than on the pre-test consultation, suggesting that the inclusion of patients’ conventional medical history was not the only variable influencing the nature of the consultations.

As mentioned above, one finding of Slade et al. was that conventional doctors tended to ask Wh-Qs more frequently than TCM practitioners [63]. They further proposed that open questions such as those in the Wh-Q format can elicit more information from patients. From the analysis of TCM consultations in this study, however, it was apparent that the TCM practitioners preferred to ask Y/N-Qs, marking this as one of the distinctions between conventional medicine and TCM consultations. This could be explained by the TCM practitioners already formulating presuppositions in their mind regarding their patients’ symptoms. For example, Kwan asked the patient ‘Do you feel dry in your mouth?’, ‘Do you have a headache?’, ‘Do you sleep well?’, ‘Do you exercise every day?’, and ‘Do you eat little meat?’; many of these questions involve presuppositions, but they can help to elicit answers that might relate to the patients’ symptoms. This reflects a difference between TCM practitioners and conventional doctors. Our findings addressed the current research gap by exploring how TCM practitioners communicate with patients receiving both TCM treatment and conventional medicine [68]. While in Hong Kong hospital system, conventional medicine used to dominate for medical treatments, this leads to questions about whether TCM and conventional medicine are complementary for treating same patient. Our findings should support future studies of communication priorities in the care of these patients, in addition to receiving both TCM and conventional medicine [27].

Several limitations can be found in this study. For examples, this study is limited to Hong Kong, and TCM practitioner–patient interactions might have different patterns in other geographical regions. Only eight TCM practitioners were recruited for this study, and they were all from Hong Kong, where TCM is outside of public hospital system and thus the findings of TCM communication practice could be different from other Chinese contexts. Due to the small sample size, generalisation of the research findings should be made cautiously.

## Conclusion

From our analysis of TCM practitioners’ consultations with their patients after a training intervention, we demonstrate that the inclusion of patients’ conventional medical histories can be beneficial in prompting TCM practitioners to ask more Wh-Qs and psychosocial questions. This could produce a more holistic understanding of patients, contribute to their satisfaction towards the treatment and provide a more patient-oriented service [3]. Pedagogically, the findings from this study can benefit not only frontline TCM practitioners but also students of TCM by enabling them to appreciate the versatility of TCM consultation discourse. Besides inquiring into the cause of patients’ discomfort, TCM practitioners also need to cultivate a conversational structure with patients that enables a mutual understanding of the problems they are encountering [5, 69–71]. Small talk has been found to lead to patients revealing useful facts about their health condition and social life [5]. Therefore, by being more familiar with the various interrogative strategies in patient communication for eliciting more information at both the medical and interpersonal levels, practitioners can achieve a more holistic and patient-centred treatment plan.

The potential impact of this study is multi-pronged. The analysis enriches our understanding of the discourse patterns of TCM communication, thereby contributing to the new knowledge of better way for TCM practitioners to communicate with patients by taking their conventional medical history. The results of integrating patients’ conventional medical histories into TCM consultations provide TCM practitioners with insights that can help them to deliver patient-centred and supportive care. The results of the study could also inspire future research into the approaches for medical practitioners to cultivate a more holistic understanding of patients’ conditions in the family medicine and TCM context. The findings shed light on how interpersonal relationships between TCM practitioners and patients can be constructed after communication training to better care for patients’ psychological concerns in addition to their physical needs.

The findings from this study will inform the training programmes of future TCM practitioners, extend their understanding of communication in acute situations, and provide a firm evidence-based foundation upon which to develop communication strategies and interventions that improve their practices. TCM practitioners can learn the communication strategies in facilitating their patients to navigate between TCM and conventional medical care in their journey. These findings will benefit conventional medical doctors and caregivers communicating with their patients using TCM terminology. More family physicians trained in evidence-based complementary medicine will become optimal integrators of holistic patient-centred care.

## Data Availability

All data generated or analysed during this study are included in this published article.

## Footnotes

1 For the purpose of this study, the number of turns refers to the practitioners’ utterances only. As all TCM consultations involve two individuals – a practitioner and a patient – the number of turns in the overall consultation was double the number indicated here (i.e. 13 turns uttered by the practitioner means that the consultation was 26 turns long including the patients’ utterances).
